# Biodiversity Patterns and DNA Barcode Gap Analysis of COI in Coastal Lagoons of Albania

**DOI:** 10.3390/biology13110951

**Published:** 2024-11-19

**Authors:** Mariola Ismailaj, Francesco Zangaro, Valeria Specchia, Franca Sangiorgio, Francesca Marcucci, Hajdar Kiçaj, Alberto Basset, Maurizio Pinna

**Affiliations:** 1 Department of Biological and Environmental Sciences and Technologies, DiSTeBA, University of Salento, Via Monteroni 165, 73100 Lecce, Italy; mariola.ismailaj@univlora.edu.al (M.I.); franca.sangiorgio@unisalento.it (F.S.); francesca.marcucci@unisalento.it (F.M.); alberto.basset@unisalento.it (A.B.); maurizio.pinna@unisalento.it (M.P.); 2 Department of Biology, University of Rome Tor Vergata, Via Ricerca Scientifica 1, 00133 Rome, Italy; 3 Department of Biology, Faculty of Technical and Natural Sciences, University “Ismail Qemali” Vlore, L. Pavarësia, Rr. Kosova, 9400 Vlorë, Albania; hajdar.kicaj@univlora.edu.al; 4 National Biodiversity Future Center (NBFC), 90133 Palermo, Italy; 5 LifeWatch Italy, LifeWatch Service Centre via Prov. Lecce-Monteroni, 73100 Lecce, Italy; 6 Research Centre for Fisheries and Aquaculture of Aquatina di Frigole, DiSTeBA, University of Salento, Via Negri, 73100 Lecce, Italy

**Keywords:** morphological identification, DNA-based methods, COI barcode, gap analysis, genetic libraries

## Abstract

Aquatic biodiversity includes a variety of unique species, their habitats, and their interactions with each other. Albania has a large hydrographic network including rivers, lakes, wetlands and coastal marine areas, contributing to a high level of aquatic biodiversity. Currently, evaluating aquatic biodiversity relies on morphological species identification methods, but DNA-based taxonomic identification could improve the monitoring and assessment of aquatic ecosystems. This study aims to evaluate the coverage of COI DNA barcodes in the reference libraries for the known aquatic animal species present in the coastal lagoons of Albania. In this study, the six most studied coastal lagoons of Albania were selected. Species data were gathered from the scientific literature and publicly available sites and studies. The collected species lists were taxonomically standardised using global public taxonomic databases like WORMS. The standardised lists were used to analyse the barcode gap of COI based on two public DNA barcode libraries: Barcode of Life Data Systems (BOLD) and NCBI GenBank. The results show that the COI DNA barcode gap in the coastal lagoons of Albania ranges from 7% (Lagoon of Patok) to 33% (Karavasta Lagoon). Fishes and Amphibia represent the groups with the lowest barcode gap (8% each), while Annelida shows the highest (47%). In conclusion, the COI gene marker for DNA-based biodiversity assessments is reliable for the coastal lagoons of Albania.

## Simple Summary

High levels of biodiversity characterize the aquatic ecosystems of Albania including coastal lagoons. They are among the most biologically rich and productive environments worldwide. These ecosystems are dynamic, transitioning between land and sea, and they serve as crucial interfaces for various ecological functions and biodiversity requiring specific monitoring programs. The current study represents a pivotal contribution to understanding the biodiversity patterns in the coastal lagoons of Albania. Species data were collected from scientific literature, grey literature, and scientific project reports. These sources about the biodiversity of Albanian lagoons rely upon traditional methods based on morphological species identification. In recent years, new innovative DNA-based methods have been developed to assess aquatic biodiversity. The efficiency of these methods is related to the availability of species “DNA barcodes” in specific international databases. In this study, we focused on the analysis of the extent of records for DNA barcodes in reference libraries, for the aquatic species of Albanian coastal lagoons. Our results reveal an overall gap of 20%, which indicates the concrete possibility of using molecular methods to investigate the marine biodiversity in Albanian lagoons.

## 1. Introduction

Aquatic biodiversity is represented by several unique species, habitats, and interactions between each other and their environment. Albania is well known for its hydrographic network, composed of rivers, lakes, wetlands, groundwaters, and coastal marine areas. These ecosystems are threatened by different anthropogenic factors, leading to biodiversity loss and habitat degradation [1]. Biodiversity conservation is based on species monitoring and maintaining richness and population size [2]. The description of changes in marine coastal waters, freshwaters, and transitional waters is based on the occurrence monitoring of target groups which are important for evaluating the conservation status of these ecosystems [3,4,5,6]. Actually, the estimation of aquatic ecosystems mainly relies upon traditional methods based on morphological species identification [7,8,9,10]. Such methods have some limitations, like the difficulties in identifying species at different stages of their life cycle (e.g., the larval stage), the need for very high expertise, and the time requirements. Also, damaged organisms can be hard to identify on a morphological basis, and the misidentification of newly introduced non-indigenous species (NIS) can occur. Due to such limitations, morphological species identifications are often limited at the genus or family level [8,10,11,12].

New innovative methods were developed for the biomonitoring of aquatic ecosystems. These methods use DNA fragments for species identification, such as DNA barcoding, DNA metabarcoding, and environmental DNA (eDNA) metabarcoding [13,14]. The application of molecular methods shows different advantages. It provides information for a large number of species at the same time and allows species identification at immature stages. Also, it allows the identification of damaged organisms, is time and cost-effective, and allows for the early warning of NIS in the studied environments [14,15,16,17,18]. Furthermore, molecular methods, e.g., eDNA metabarcoding, permit biodiversity assessments without harming organisms [19,20]. Recent advances in High Throughput Sequencing (HTS) technology increased the ability for rapid large data biomonitoring [21]. However, the applicability and accuracy of DNA-based taxonomic identification largely depend on the DNA barcode reference libraries’ taxa coverage and the quality of the available barcode records [13,22]. So, to efficiently apply such methods, it is essential to know the existing gap in the DNA barcode reference libraries, such as the Barcode of Life Data Systems (BOLD) [23] and NCBI GenBank [24]. 

The mitochondrial Cytochrome c Oxidase subunit 1 (COI) gene marker has been widely used for the barcoding of animal species and, thus, is largely used in DNA-based biodiversity assessments and biomonitoring [25,26,27]. More than 8.3 million and 3.7 million COI reference sequences have been recorded in the BOLD and GenBank databases, respectively (up to March 2021) [23,24]. Importantly, even other barcode regions (e.g., the 12S rRNA gene marker and the 18S rRNA gene marker) are used for DNA barcoding, metabarcoding, and eDNA metabarcoding, and may represent better gene markers for some taxa. Many studies concerning the gaps in the DNA barcode reference libraries for aquatic species vary between taxon groups in different EU countries. In reference libraries, 60% of the taxa have less than five barcodes recorded [13]. This gap may be higher in developing countries, where there is still a lack of DNA barcoding of aquatic species, and Albania may be one of those [10]. 

In this study, we focused on the analysis of the extent of records for COI DNA barcodes in DNA barcode reference libraries for the aquatic animal species of Albanian coastal lagoons. As Albania has several aquatic ecosystems, it even has many aquatic species, accounting for 20–23% of all EU aquatic species, including some endemic and endangered ones [25]. For this analysis, we considered Albania’s six major and most studied coastal lagoons: Kune-Vain Lagoon, Patok Lagoon, Karavasta Lagoon, Narta Lagoon, Orikumi Lagoon, and Butrinti Lagoon. The first step was preparing a species checklist for the six coastal lagoons. After this, we standardised the taxonomy based on international species databases like the World Register of Marine Species (WORMS; https://www.marinespecies.org accessed on 25 January 2023), the Global Biodiversity Information Facility (GBIF; https://www.gbif.org accessed on 25 January 2023), and FishBase (https://www.fishbase.se accessed on 25 January 2023). Then, we performed the first gap analysis of all COI records in two barcode reference libraries, BOLD and NCBI GenBank, focusing on aquatic animal species in Albanian coastal lagoons. 

The aims of this study are as follows: (1) to describe the already known biodiversity in the coastal lagoons of Albania; (2) to analyse the current status of COI records of aquatic animal species in Albanian coastal lagoons; (3) to compare the barcode coverage of different aquatic animal groups in two reference databases (BOLD and NCBI GenBank); and (4) to reveal the barcode record differences in the coastal lagoons of Albania. 

## 2. Material and Methods

### 2.1. Coastal Lagoons Selection and Species Lists Search 

**Figure 1:**
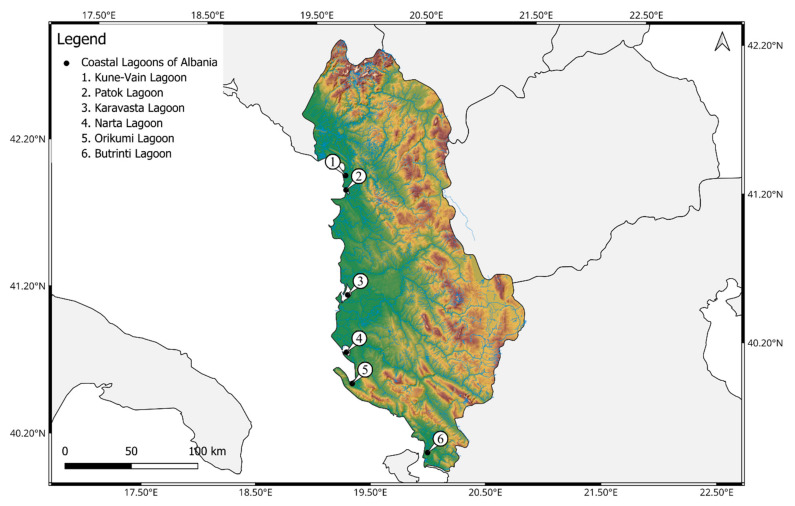
The six investigated coastal lagoons of Albania: 1. Kune-Vain Lagoon; 2. Patok Lagoon; 3. Karavasta Lagoon; 4. Narta Lagoon; 5. Orikumi Lagoon; and 6. Butrinti Lagoon. The map was generated using QGIS v3.32.0 (https://qgis.org accessed on 2 September 2024).

In this investigation, 6 coastal lagoons of Albania were selected as follows: Kune-Vain Lagoon, Patok Lagoon, Karavasta Lagoon, Narta Lagoon, Orikumi Lagoon, and Butrinti Lagoon (Figure 1).

These coastal lagoons host high levels of biodiversity and provide high rates of ecosystem goods and ecosystem services. They are used for fisheries, agriculture, truism, fish farming, and salt production.

A total species list was prepared for each of the selected ecosystems. Such information was collected by searching and downloading scientific literature such as scientific articles, national and international project outcomes, and scientific data from national institutions like the Ministry of Environment, Protected Areas Agencies, and Universities. For the scientific literature search, multiple databases related to cross-disciplinary research were queried, such as Scopus (https://www.scopus.com accessed on 1 September 2022), Google Scholar (https://scholar.google.com accessed on 1 October 2022), Science Direct (https://www.sciencedirect.com accessed on 1 November 2022), and Web of Science (https://www.webofscience.com accessed on 1 December 2022). The databases were queried using different combinations of keywords including “ecosystem name”, “biodiversity”, “species list”, and “species checklist”. 

A species list for every ecosystem was built according to the related scientific literature retrieved. The species lists were organised based on the Darwin-Core classification system, and only aquatic animal species were selected for the COI DNA barcode gap analysis. After the species list classifications, the taxonomy was standardised using databases like WORMS, GBIF, and FishBase. This process allowed us to overcome issues related to synonyms and unaccepted species names. 

According to the scientific literature retrieved for the coastal lagoons of Albania, the main available environmental characteristics were collected for each of the six considered coastal lagoons of Albania. The data collected from the different sources allowed the evaluation of the state of the art of biodiversity in the selected coastal lagoons. The number of species in each ecosystem was calculated and a multivariate Principal Component Analysis was performed, with the data matrix consisting of the environmental parameters as independent variables and the number of species as the responsive variable. All calculations were made using the R Software (v4.3.1). Specifically, the factoextra and ggplot2 packages were used for PCA computation and visualization.

### 2.2. Study Areas

In this study, the 6 major coastal lagoon ecosystems from northern to southern Albania were considered as follows: Kune-Vain Lagoon, Patok Lagoon, Karavasta Lagoon, Narta Lagoon, Orikumi Lagoon, and Butrinti Lagoon (Figure 1).

#### 2.2.1. Butrinti Lagoon

Butrinti Lagoon is a tectonic lagoon extending for 16 km^2^. It is surrounded by forested hills and mountains and encompassed by freshwater and saltwater marshlands. It is a highly productive ecosystem (mesotrophic to hypertrophic), favourable for fishing and aquaculture (e.g., mussel growth). The oxygenated layer offers favourable habitats for shellfish reproduction. It is well known for Mediterranean mussel (*Mytilus galloprovincialis*) farming. However, the lagoon conditions and the mussel growth can be disturbed by Harmful Algal Bloom (HAB) events by toxic phytoplanktonic species [28].

#### 2.2.2. Karavasta Lagoon

With a surface area of 45 km^2^ and an average depth of 1.5 m, Karavasta Lagoon is the widest lagoon in Albania. It is part of the Divjake-Karavasta National Park, one of the most important areas for its ecological values [29]. This coastal lagoon hosts a large number of species. Also, it is surrounded by a pine forest hosting a large group of flora and fauna species. The most common and frequently observed species are *Sparus aurata*, *Mugil cephalus*, *Solea vulgaris*, *Belone belone*, *Dicentrarchus labrax*, *Gobius bucchichi*, members of the Cyprinodontidae family, *Atherina boyeri*, and *Syngnathus* sp. Some are engendered by overfishing [30]. Also, the loggerhead turtle (*Caretta caretta*) casually visits the coastal waters in this area. The indigenous Aleppo pine (*Pinus halepensis*) and the Umbrella pines (*Pinus pinea*) dominate the flora. Woodland develops between the dune slacks, alongside the broad wet depressions or on the edge of the slacks. Eels (*Anguilla anguilla*) contribute the biggest share of the income from the small-scale fishery, followed by sea bass, gilt-head bream, flatfish, and mullets. Finally, it is well known for the number of birds, as it is a Ramsar Protected Area. For instance, birds like *Pelecanus crispus* can be observed in this lagoon [29].

#### 2.2.3. Kune-Vain Lagoon

Kune-Vain Lagoon is located near Lezha city, in the north of Albania. This is the first protected area in Albania. It has a surface area of 30 km^2^ and consists of two sites: Kune and Knalla wetland in the northern part of the Drini delta, and Merxhani and Vain lagoon in the South. This site communicates with the Drini River by an artificial channel and Merxhani Lagoon communicates with the sea by a central channel [28]. The average depth goes from 0.7 m to 1.3 m. It has a high diversity of phytoplanktonic species. It is one of the wetlands also known as an important wintering site for birds like the endangered *Phalacrocorax pygmaeus*. Other rare wintering birds in these marine and coastal habitats are dabbling and diving ducks [31].

#### 2.2.4. Narta Lagoon

Narta Lagoon is located in the northern part of Vlora Bay. It is one of the most important Albanian wetlands [28,32]. Narta Lagoon is a shallow coastal lagoon bordered by hills to the south, the Saline to the north, and wetlands to the west. The average depth varies from 1.3 m to 2.1 m with a surface area of 41.8 km^2^. The lagoon communicates with the sea by 2 arterial channels. The mean evaporation rate is about 1260 mm per year. The temperature goes from 5 °C to 25 °C and the salinity goes from 28% during winter to 75% in summer. During summer, the surface area decreases by about 30% [28]. As part of the Protected Landscape Pishe Poro-Narta, this lagoon is known for hosting the highest rate of biodiversity. The Narta Lagoon ecosystem is an important complex of international importance for the species it hosts, especially birds. The area fulfils the criteria of the Ramsar Convention [31]. Narta Lagoon is classified as a Geo-monument of international importance [33] and a Special Protected Area for birds [34].

#### 2.2.5. Orikumi Lagoon

Orikumi Lagoon is a small coastal lagoon extending for 1.5 km^2^ in the Area of Vlora, near the Karaburun-Sazan National Marine Park. It is well known as a rich habitat for fish species. The most important fish species in this site are *Sparus aurata*, *Mugil cephalus*, *Anguilla anguilla*, and *Dicentrarchus labrax*. Professional fishing exists along the coast of Rreza-Karaburuni and Sazani. It is an important coastal lagoon even for waterbirds, as 105 species are known to inhabit this area. More than 60 species are residents [28].

#### 2.2.6. Patok Lagoon

Patok Lagoon is located between the Mati River in the north and the Ishmi River in the South. The surface area is 4.8 km^2^. This lagoon is characterised by a high vegetal and animal species diversity. It is one of the most important sites for fishing. It hosts a large number of species, like molluscs and fishes. Patok Lagoon is well known for the high number of loggerhead turtles (*Caretta caretta*) visiting this area. Patok Lagoon represents an important site for endangered bird species like *Pelecanus crispus*, *Phalacrocorax pygmaeus* and *Ciconia ciconia* [28].

### 2.3. COI DNA Barcode Research in Reference Libraries

The target gene selected for the gap analysis is the COI gene marker, as it represents one of the most barcoded genes for animal species and shows the largest coverage in the DNA barcode reference libraries [25,26,27].

After the taxonomy revision for all the species lists constructed for the selected coastal lagoons, the COI DNA barcode gap analysis was performed. Specifically, all the aquatic animal species names were singularly queried in the international DNA barcode reference libraries BOLD and NCBI GenBank [23,24].

For each species, the number of available COI DNA barcodes was registered. The gap was calculated as the percentage of species without a COI DNA barcode in each of the selected coastal lagoons. The gap was calculated for each coastal lagoon and each taxonomic group. All calculations were made using the software Microsoft Excel.

## 3. Results

### 3.1. Known Biodiversity in the Coastal Lagoons of ALBANIA

The scientific literature from 1996 to 2022 retrieved for the six investigated coastal lagoons was consulted to obtain information about each lagoon’s surface area, the number of sources available for each lagoon, and the total species diversity inhabiting these ecosystems. For each lagoon, average values of chlorophyll concentration, depth, dissolved oxygen, pH, salinity, temperature, and total suspended solids were also collected. Some general information for each considered coastal lagoon is summarised in Table 1.

The lagoon with the highest number of representative species is Narta Lagoon (675 species), followed by Karavasta Lagoon (509 species) and Butrinti Lagoon (442 species). Karavasta Lagoon has the largest surface area (45 km^2^), followed by Narta Lagoon (41.8 km^2^). Orikumi Lagoon hosts the lowest number of species (209 species). Also, Orikumi Lagoon is the smallest (1.5 km^2^ of surface area) and is the least studied, as only five sources were retrieved after the bibliographic search. Narta Lagoon is the most studied, with 17 retrieved sources after the bibliographic search.

### 3.2. Statistical Correlations

**Figure 2:**
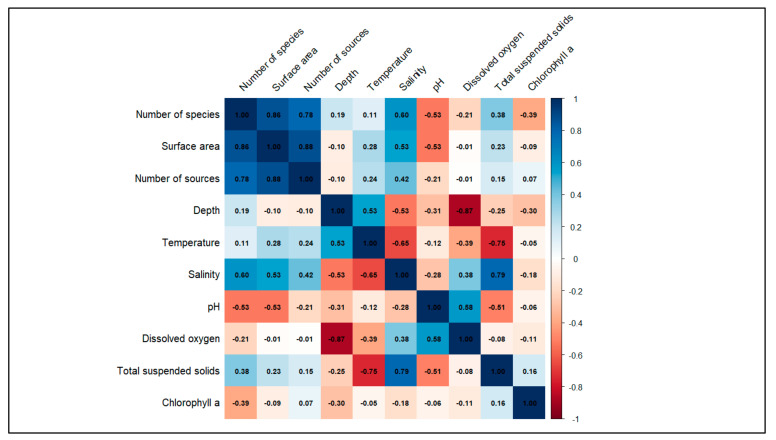
Correlation matrix of number of species and other considered variables for the coastal lagoons of Albania.

**Figure 3:**
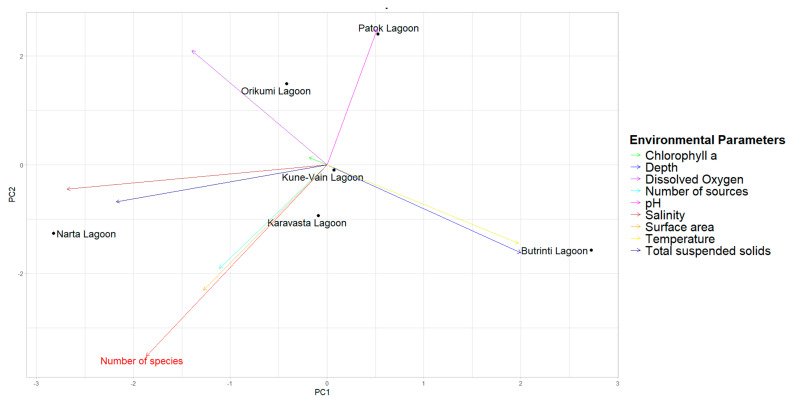
Principal Component Analysis (PCA) biplot for the number of species (in red) and other considered variables for the coastal lagoons of Albania.

According to the analysed data, a multivariate approach displaying the correlation matrix of species and environmental parameters (Figure 2), and the Principal Component Analysis (PCA) biplot (Figure 3) was conducted. In Figure 2, the correlation matrix provides a visual summary of the relationships between the number of species and the other considered variables for the coastal lagoons of Albania. In particular, the matrix shows a positive correlation between the number of species and surface area, between the surface area and number of sources, and between the number of species and number of sources. Also, a moderated correlation between the number of species and salinity is displayed.

In Figure 3, the PCA biplot better explains the correlations between the number of species and other variables in the coastal lagoons of Albania. In particular, this figure displays more clearly the strong correlation between the number of species, surface area, and number of studies. These results suggest that larger lagoons tend to support higher species richness, as well as to represent preferred study areas for biodiversity studies [91].

### 3.3. COI DNA Barcode Gap Analysis Results

**Figure 4:**
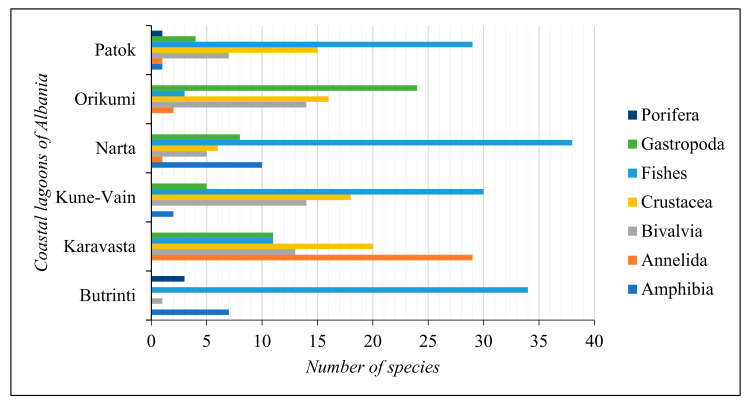
Number of species per taxonomic group in the coastal lagoons of Albania.

For the gap analysis, only the aquatic animal species reported in all the Albanian coastal lagoons were considered. The main aquatic animal taxonomic groups characterising the coastal lagoon ecosystems of Albania are Amphibia, Annelida, Bivalvia, Crustacea, Fishes, Gastropoda, and Porifera. The composition and abundance of these taxonomic groups in the coastal lagoons of Albania are reported in Figure 4.

Fishes are the most represented taxonomic group in all the coastal lagoons but in Karavasta Lagoon, where the most represented taxonomic group is Annelida.

**Figure 5:**
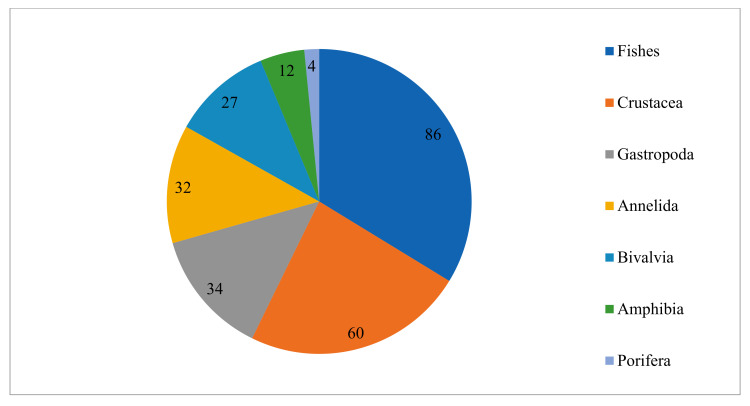
Total representative species per taxonomic group.

The chart of the number of species per taxonomic group (Figure 5) shows that Fishes (86) are the most represented taxonomic group in the total aquatic species list, followed by Crustacea (60) and Gastropoda (34). The least represented taxonomic groups are Amphibia (12) and Porifera (4).

**Figure 6:**
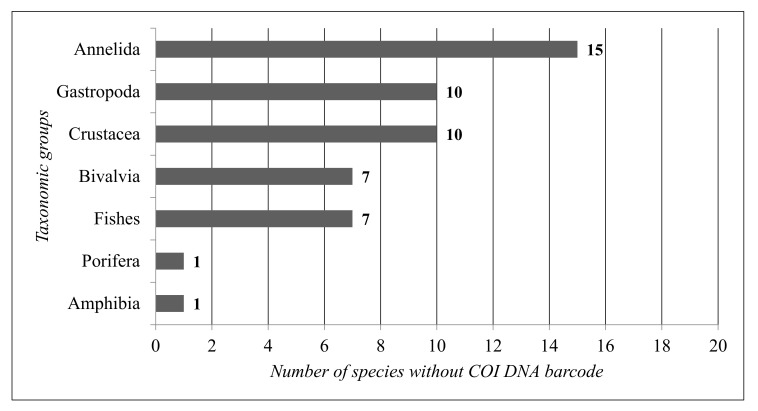
Number of species without a COI DNA barcode per taxonomic group.

For the COI DNA barcode gap analysis, only the aquatic animal species were considered, as COI is well represented in the DNA barcode reference libraries. Based on the data concerning the COI barcodes for the main taxonomic groups reported in all the coastal lagoons, the total calculated COI barcode gap is 20% (Figure 6). 

The taxonomic groups with the lowest COI gap are Fishes (8%) and Amphibia (8%), while the taxonomic group with the highest COI gap is Annelida (47%).

**Figure 7:**
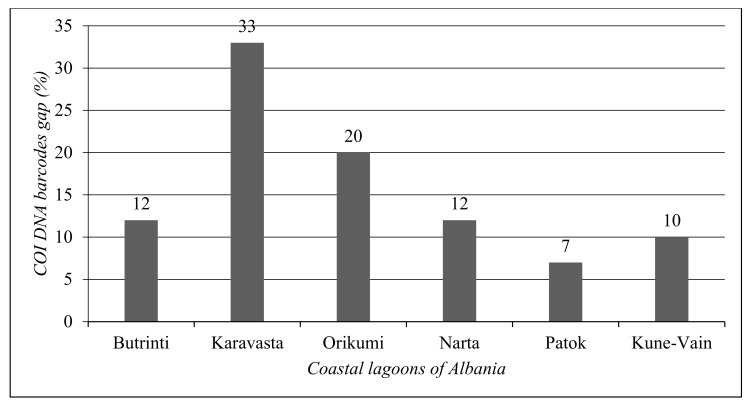
Total COI DNA barcode gap for each coastal lagoon of Albania.

In Figure 7, the COI gap was reported for each coastal lagoon ecosystem. Karavasta Lagoon shows the largest gap in the COI DNA barcode (33%), followed by Orikumi Lagoon (20%). Patok Lagoon shows the lowest COI DNA barcode (7%).

**Figure 8:**
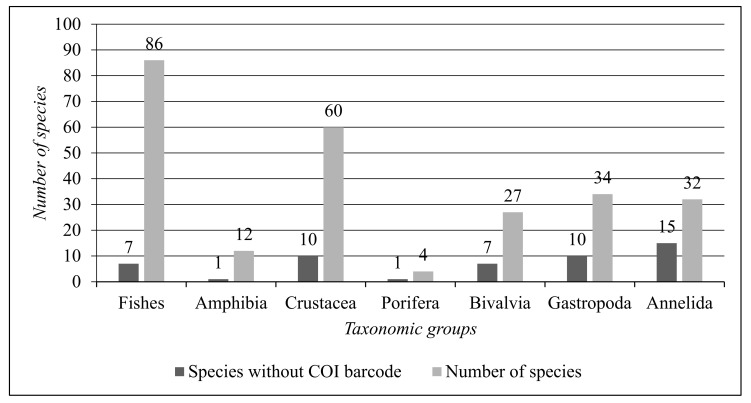
Comparison between the total number of aquatic animal species per taxonomic group and the number of species without a COI DNA barcode.

Based on the results concerning the COI DNA barcode gap analysis of the analysed species, Figure 8 synthesises the comparison between total aquatic animal species per taxonomic group and the number of species without a COI barcode in the DNA barcode reference libraries.

According to the results (Figure 6, Figure 7 and Figure 8), the most represented taxonomic groups are Fishes (86 species) and Crustacea (60 species). The taxonomic groups with the highest numbers of species without a COI DNA barcode are Annelida (15 species) and Gastropoda (10 species). The representative species of Amphibia are twelve, and the Amphibia species without a COI barcode is only one. Fishes have a low COI DNA barcode gap in the reference libraries, with only seven species out of eighty-six.

### 3.4. COI Barcode Gap for Each Coastal Lagoon Ecosystem

**Figure 9:**
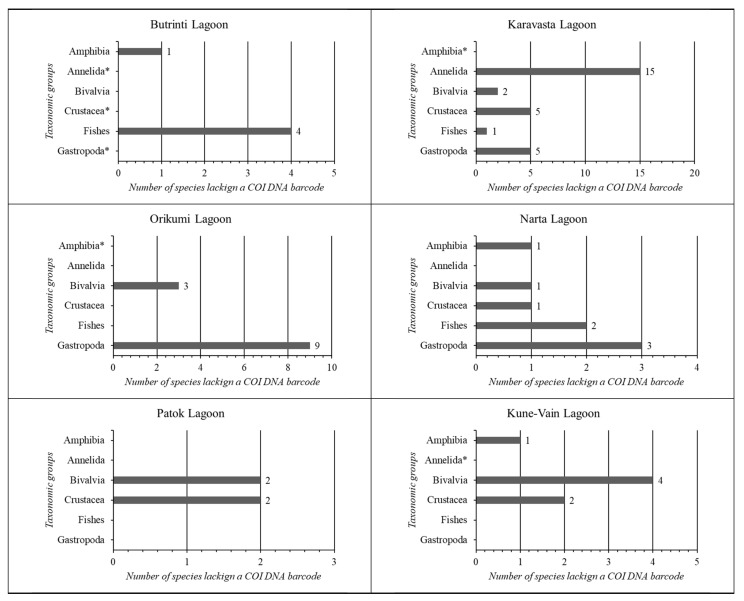
Summary of the COI DNA barcodes missing for each taxonomic group in each coastal lagoon ecosystem of Albania. * Taxonomic group absent from the general species checklist.

In Figure 9, the COI DNA barcodes missing for each taxonomic group in each coastal lagoon ecosystem of Albania were synthesised.

The analysis of each coastal lagoon shows that the lagoon with the lowest COI gap is Patok Lagoon (7%), followed by Kune-Vain Lagoon (10%). Karavasta Lagoon shows the highest gap (33%). The highest number of species without a COI DNA barcode per taxonomic group for Butrinti Lagoon belongs to Fishes (4); for Karavasta Lagoon belongs to Annelida (15); for Kune-Vain Lagoon belongs to Bivalvia (4); for Narta Lagoon belongs to Gastropoda (3); and for Orikumi Lagoon belongs to Gastropoda (9).

## 4. Discussion

The current study represents a pivotal contribution to understanding the biodiversity patterns in the coastal lagoons of Albania. The correlation matrix (Figure 2) and the PCA biplot (Figure 3) show a strong correlation between the number of species in Albanian lagoons, the lagoons’ surface area, and the research efforts in the coastal lagoons’ areas. These results provide a valuable understanding of the main variables affecting the species richness and biodiversity in Albania’s coastal lagoons. The results highlight that size and research effort are the primary drivers for explaining the number of species retrieved in the literature since it appears that larger lagoons can support higher species biodiversity, representing the preferred target areas for biodiversity monitoring studies and activities [35]. Furthermore, the results highlight that environmental factors like pH, dissolved oxygen, and salinity also play important roles in shaping the biodiversity of Albania’s coastal lagoons, underlying the importance of rapid, high throughput, and innovative biodiversity assessment studies in small and understudied coastal lagoons and ecosystems.

In light of this, our findings provide a thorough analysis of the COI DNA barcode gap for aquatic animal species in the major coastal lagoons of Albania. Our results reveal an overall gap of 20%, which, when compared to similar research conducted in Europe and China, falls within an expected range [92,93]. In China, for example, the COI DNA barcode gap for aquatic species in rivers can range from 40% to 70%, while studies in Europe have reported gaps of approximately 50% for aquatic insects. In southern Italy, an investigation about the influence of DNA barcode databases’ incompleteness on species diversity indices, ecological indicators, and ecological assessment in transitional water ecosystems of the southeast Mediterranean revealed a COI DNA barcode gap of 36% for benthic macroinvertebrates, with a difference in 27% of sites when assessing the ecological quality status by applying both morphological and molecular approaches [11]. 

The relatively lower gap observed in the Albanian coastal lagoons may be attributed to focused efforts on documenting fish species, which exhibit the smallest gap (8%) among the taxonomic groups examined.

The identified barcode gap has crucial implications for biodiversity monitoring and conservation strategies. A great portion of species, particularly within the Annelida and Gastropoda groups, lack a COI DNA barcode representation, limiting the potential for molecular methods, such as DNA barcoding or eDNA metabarcoding, to be fully effective in these taxa. This is particularly problematic for species which are integral to the ecosystem functioning of coastal lagoons due to their roles in sediment processing and trophic interactions [17]. Without comprehensive molecular records, these species can remain underrepresented in biodiversity assessments and conservation studies (Table 2).

Filling these gaps requires targeted efforts to expand reference libraries, especially for invertebrate species, which often play essential roles in nutrient cycling and habitat structure. Encouraging multidisciplinary collaborations between experts in the morphological identification of species and experts in molecular applications could enhance the efficiency of species identification and the development of barcode records.

When compared to other Mediterranean ecosystems, the coastal lagoons of Albania demonstrate relatively high levels of biodiversity, with Narta Lagoon hosting the largest number of species (675). However, the gap in COI DNA barcodes for certain taxa in Albania mirrors findings in other Mediterranean regions, where the underrepresentation of non-commercial species is common. For instance, studies in Italian lagoons have shown similar gaps in barcode representation for benthic macroinvertebrates, which are key bioindicators of water quality [11]. This pattern suggests that Albania, like other countries in the Mediterranean Sea, faces several challenges in cataloguing the full diversity of its aquatic ecosystems due to the complexity of lagoon habitats and the varied pressures they face from anthropogenic activities.

## 5. Conclusions

The coastal lagoons of Albania, with their diverse habitats and species, serve as critical areas for conservation efforts. These ecosystems support endemic and endangered species and provide essential ecosystem services such as water filtration, carbon sequestration, and nurseries for commercially important fish species. The results from this study suggest that enhancing the DNA barcode libraries, particularly for invertebrate species, could improve the efficacy of molecular methods in monitoring these vital ecosystems. Furthermore, the findings support the use of eDNA techniques as a non-invasive approach to track biodiversity changes and detect the early presence of non-indigenous species (NIS), which pose significant threats to the ecological balance of these ecosystems.

This analysis of COI DNA barcode gaps in Albanian coastal lagoons represents an important step toward a broader application of DNA-based monitoring techniques in aquatic ecosystems. However, the results also underscore the need for multidisciplinary efforts to fill in the gaps in barcode coverage, particularly for invertebrate taxa that are critical to ecosystem functioning but remain underrepresented in molecular databases. Future research should prioritise the expansion of DNA barcode libraries through collaborative initiatives that combine traditional taxonomic expertise with molecular experts. Additionally, the integration of alternative genetic markers could enhance species identification, especially for taxa with low COI variability. As molecular tools become increasingly central to biodiversity assessments, comprehensive and updated reference libraries will be essential for the accurate and effective conservation of Albanian coastal lagoons and their unique biodiversity.

## Figures and Tables

**Table 1:** General data for each coastal lagoon of Albania.

Name	Surface Area (km^2^)	Number of Species	Number of Sources
Karavasta Lagoon	45	509	15 [28,29,35,36,37,38,39,40,41,42,43,44,45,46,47]
Butrinti Lagoon	16.3	442	11 [28,30,35,48,49,50,51,52,53,54,55]
Narta Lagoon	41.8	675	18 [28,32,34,37,38,56,57,58,59,60,61,62,63,64,65,66,67,68]
Orikumi Lagoon	1.5	209	5 [35,48,69,70,71]
Patok Lagoon	4.8	241	11 [28,35,37,38,72,73,74,75,76,77,78]
Kune-Vain Lagoon	20	252	13 [28,79,80,81,82,83,84,85,86,87,88,89,90]

**Table 2:** Species without a COI DNA barcode.

Taxon	Family	Species Name
Annelida	Naididae	*Aktedrilus cuneus*
	Naididae	*Aktedrilus monospermathecus*
	Opheliidae	*Armandia cirrhosa*
	Capitellidae	*Capitella giardi*
	Naididae	*Limnodriloides maslinicensis*
	Nereididae	*Nereis rava*
	Syllidae	*Parapionosyllis minuta*
	Phyllodocidae	*Phyllodoce macrophthalmos*
	Spionidae	*Polydora ciliata*
	Dorvilleidae	*Protodorvillea kefersteini*
	Syllidae	*Salvatoria limbate*
	Dorvilleidae	*Schistomeringos rudolphi*
	Lumbrineridae	*Scoletoma laurentiana*
	Serpulidae	*Simplaria pseudomilitaris*
	Serpulidae	*Spirobranchus lamarcki*
Amphibians	Ranidae	*Rana graeca*
Bivalvia	Semelidae	*Abra segmentum*
	Carditidae	*Glans trapezia*
	Lucinidae	*Loripes orbiculatus*
	Semelidae	*Scrobicularia cottardii*
	Solenidae	*Solen capensis*
	Veneridae	*Venerupis corrugata*
	Veneridae	*Venerupis geographica*
Crustacea	Varunidae	*Brachynotus sexdentatus*
	Carcinidae	*Carcinus mediterraneus*
	Leptocheliidae	*Chondrochelia savignyi*
	Corophiidae	*Corophium orientale*
	Anthuridae	*Cyathura carinata*
	Callianassidae	*Gilvossius tyrrhenus*
	Harpacticidae	*Harpacticus gracilis*
	Sphaeromatidae	*Lekanesphaera monodi*
	Aoridae	*Microdeutopus gryllotalpa*
	Ectinosomatidae	*Microsetella norvegica*
Osteichthyes	Bothidae	*Arnoglossus imperialis*
	Cyprinidae	*Barbus peloponnesius*
	Cyprinidae	*Chondrostoma nasus*
	Gobiidae	*Knipowitschia goerneri*
	Soleidae	*Solea solea*
	Cyprinidae	*Squalius pamvoticus*
Gastropoda	Cerithiidae	*Cerithium vulgatum*
	Pyramidellidae	*Megastomia conoidea*
	Assimineidae	*Paludinella sicana*
	Rissoidae	*Pusillina lineolata*
	Rissoidae	*Pusillina marginata*
	Rissoidae	*Pusillina radiata*
	Rissoidae	*Rissoa membranacea*
	Rissoellidae	*Rissoella opalina*
	Neritidae	*Smaragdia viridis*
	Pyramidellidae	*Spiralinella incerta*
Porifera	Chalinidae	*Chalinula renieroides*

## Data Availability

All data are reported in the paper.
